# Identifying Regulators for EAG1 Channels with a Novel Electrophysiology and Tryptophan Fluorescence Based Screen

**DOI:** 10.1371/journal.pone.0012523

**Published:** 2010-09-02

**Authors:** Tinatin I. Brelidze, Anne E. Carlson, Douglas R. Davies, Lance J. Stewart, William N. Zagotta

**Affiliations:** 1 Department of Physiology and Biophysics, University of Washington School of Medicine, Seattle, Washington, United States of America; 2 Emerald BioStructures, Inc., Bainbridge Island, Washington, United States of America; University of California, United States of America

## Abstract

**Background:**

*Ether-à-go-go* (EAG) channels are expressed throughout the central nervous system and are also crucial regulators of cell cycle and tumor progression. The large intracellular amino- and carboxy- terminal domains of EAG1 each share similarity with known ligand binding motifs in other proteins, yet EAG1 channels have no known regulatory ligands.

**Methodology/Principal Findings:**

Here we screened a library of small biologically relevant molecules against EAG1 channels with a novel two-pronged screen to identify channel regulators. In one arm of the screen we used electrophysiology to assess the functional effects of the library compounds on full-length EAG1 channels. In an orthogonal arm, we used tryptophan fluorescence to screen for binding of the library compounds to the isolated C-terminal region.

**Conclusions/Significance:**

Several compounds from the flavonoid, indole and benzofuran chemical families emerged as binding partners and/or regulators of EAG1 channels. The two-prong screen can aid ligand and drug discovery for ligand-binding domains of other ion channels.

## Introduction

The EAG family of voltage-gated K^+^ channels is encoded by genes KCNH1-8 [1]. EAG family channels cluster by sequence similarity into three subfamilies: ether-à-go-go (EAG), eag-related gene (ERG) and eag-like K^+^ channel (ELK) [2]. For the most part, EAG1 expression is confined to the central nervous system [3]. However, EAG1 mRNA is also expressed in several cell lines derived from cancerous tumors and in most types of human primary cancer studied so far [4], [5], [6], [7], [8]. Cells exogenously expressing EAG1 channels display properties seen in cancerous cells, including aggressive tumor formation when injected into immune-depressed mice [8]. Further, pharmacologic blockade of EAG1 channel activity [9], [10], [11] and inhibition of channel expression by RNAi interference [12] decreased cell proliferation in tumor tissues.

**Figure 1:**
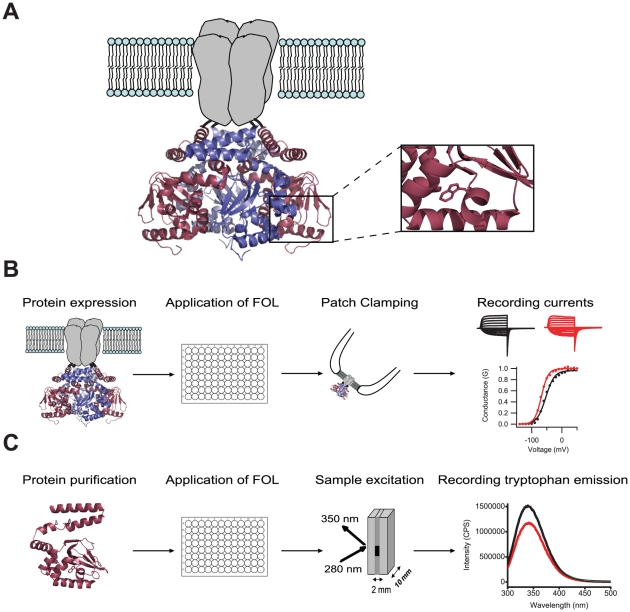
Schematic of the two-pronged screen designed to identify compounds that bind and regulate EAG1 channels. (A) EAG1 channel subunits form tetramers around a central, K^+^ conducting pore. The ribbon representation is a homology model of mEAG1#505-702, obtained with SWISS-MODEL based on the crystal structure of the carboxy-terminal region of mHCN2 [42]. Enlarged view on the right shows a tryptophan residue at position 649 in the CNBD employed as a reporter of ligand binding. (B) For the electrophysiology arm of the screen, mEAG1 channels were expressed heterologously and the FOL pools were applied to inside-out patches containing these channels. The currents from EAG1 channels were recorded in the absence (black), presence (red) and after washout of FOL compounds. The conductance-voltage relationship was determined from the tail currents and normalized to the maximal conductance in the absence of drug. (C) For the fluorescence portion of the screen, mEAG1#505-702 was expressed and purified from bacteria. The FOL compounds were applied to mEAG1#505-702 in solution, the sample was placed in a quartz cuvette and excited at 280 nm wavelength. The mEAG1#505-702 emission spectra were recorded in the absence and presence of the FOL compounds (black and red traces, respectively).

Channels in the EAG family share a basic architecture with most voltage-gated K^+^ channels. They have four subunits arranged to form a central, K^+^ conducting pore (Fig. 1A). In addition, each subunit has large intracellular amino- and carboxy- terminal regions. The amino-terminal region contains a Per-Arnt-Sim (PAS) domain [13]. In other proteins, PAS domains serve as signaling modules that bind to small molecules and other proteins [14]. The carboxy-terminal region contains a cyclic nucleotide-binding domain (CNBD) that shares sequence similarity with the CNBD of the cyclic nucleotide-gated (CNG) channels, and hyperpolarization-activated cyclic nucleotide-modulated (HCN) channels (Fig. 1A) [2], [15]. Despite having a CNBD, vertebrate EAG family channels are not regulated by the direct binding of cyclic nucleotides [16], [17]. Because they contain known binding motifs but no known ligands, we classify EAG1 channels as orphan receptors.

We sought to deorphanize the EAG1 channel by identifying compounds that regulate the channel and bind to the CNBD. In this study we developed a novel, two-pronged approach to screen a chemical library for EAG1 channel regulators. The compounds in the library were simultaneously screened for functional effects on the EAG1 channels using inside-out patch-clamp recordings, and for binding to the CNBD using tryptophan fluorescence. The combined use of two orthogonal screens greatly increases the chances of identifying a specific channel regulator. With this two-pronged screen we uncovered several regulators and binding partners for EAG1 channels that cluster into three distinct chemical families: flavonoids, indoles, and benzofurans.

## Results

To identify EAG1 channel regulators, we screened a version of the Fragments of Life (FOL) chemical library (Emerald BioStructures, Bainbridge Island, WA) against EAG1 channels with electrophysiology and fluorescence based methods. The core of the FOL library consists of 218 metabolites, and derivatives thereof, involved in diverse metabolic pathways and produced by various living organisms. The FOL library used in our study contained 768 small biologically relevant molecules grouped into 96 pools of seven to ten compounds. The distribution of the library compounds into pools allowed for a more efficient method of library screening. The FOL library was created based on knowledge of protein structures and their cognate metabolite-binding partners for structure-based drug discovery, and was previously used to identify modulators of leukotriene A4 hydrolase [18]. Here we implemented a novel application of the FOL library as a screen for EAG1 channel ligands.

### Screening the FOL library with electrophysiology

To screen for functional effects on the full length EAG1 channels, pools of the FOL compounds were applied to membrane patches expressing EAG1 channels. The EAG1 currents were recorded in the inside-out configuration of the patch-clamp technique in order to expose the intracellular putative ligand binding domains to the library compounds. Currents were recorded in response to depolarizing voltage steps in the absence, presence, and after washout of FOL compounds (Fig. 1B, see Materials and Methods). The currents were then analyzed to determine the effects of the FOL compounds on the conductance-voltage relationship, and the kinetics of activation and deactivation. In the first round of screening, we tested 96 pools each containing seven to ten FOL compounds. Pools that were identified as ‘hits’ were further studied by examining the effects of their individual compounds on channel gating.

### Screening the FOL library with tryptophan fluorescence

In parallel with the electrophysiology screen, binding of the FOL compounds to the isolated C-terminal region of EAG1 channels containing both the C-linker and CNBD, mEAG1#505-702, was probed with a fluorescence based screen. This screen employs tryptophan fluorescence, which is environmentally sensitive and hence can be used to report ligand binding [19]. For example, the fluorescence intensity of a tryptophan residue introduced in the cyclic nucleotide-binding pocket of the HCN2 channel at position 586, decreased upon binding of cyclic-nucleotides in a concentration dependent manner [16]. The mEAG1#505-702 has two endogenous tryptophan residues: at position 649, analogous to 586 in HCN2 channels (enlarged view in Fig. 1A), and at position 544 in the C-linker. The fluorescence of these tryptophan residues could change upon ligand binding to the isolated C-terminal region of EAG1 channel, as observed in HCN2 channels with cyclic nucleotides. The data acquisition procedure is summarized in Fig. 1 and is described in more detail in Fig. S1. In each experiment, the fluorescence emission spectra of mEAG1#505-702 were recorded in a quartz cuvette before and after addition of the FOL pools (Fig. 1C and Fig. S1A). Some pools were themselves fluorescent upon excitation and/or had high optical density leading to an inner filter effect. To separate the background fluorescence of the pool from changes in the tryptophan fluorescence, the emission spectra of the pool alone was recorded (blue trace in Fig. S1A) and subtracted from that of mEAG1#505-702 recorded in the presence of the pool. The absorbance of each FOL pool was also recorded (Fig. S1B) and the inner filter effect was corrected for as described in the METHODS. The final emission spectra of mEAG1#505-702 in the presence of a FOL pool represented the inner filter corrected and background subtracted spectra (Fig. 1C and Fig. S1C).

Changes in tryptophan fluorescence intensity can result from changes in the environment of the tryptophan residue or energy transfer due to ligand binding. However changes in tryptophan fluorescence may also arise from non-specific interactions between the FOL compounds and free tryptophan in solution. To determine the non-specific effects the pools of FOL compounds were added to free tryptophan in solution. The fluorescence spectrum of the free tryptophan was recorded before and after addition of the FOL pools (Fig. S1A). Similar to the analysis for mEAG1#505-702, the free tryptophan fluorescence was background subtracted and corrected for the inner filter effect (Fig. S1A–C). The final effect of a compound was quantified as the difference in the percent fluorescence intensity change of mEAG1#505-702 and free tryptophan upon application of the pool of FOL compounds and is referred to here as the relative percent change (RPC). The pools of FOL compounds with high RPC were further studied by examining individual compounds separately.

### Overview of the screen results

**Figure 2:**
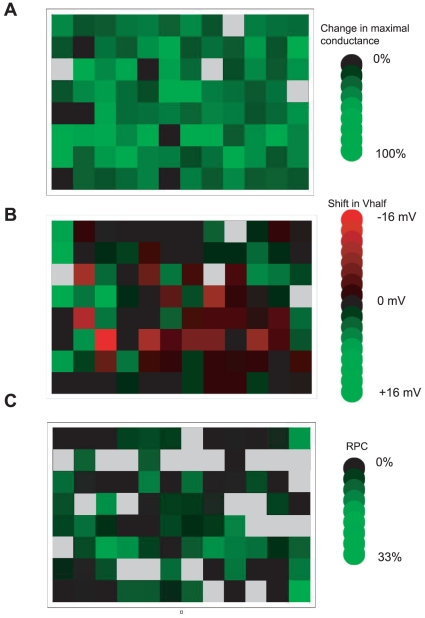
Summary of the screen results. (A) The change in relative conductance of the electrophysiology portion of the screen is plotted in a black to green color gradient, with pools that did not change the relative conductance colored black, and those with a 100% reduction in conductance colored in bright green. (B) The shift of the V_half_ of the conductance versus voltage relationship from the electrophysiology portion of the screen is plotted in a black to green color gradient, for shifts to more positive potentials indicated by shades of green (with a shift of 16 mV indicated by the brightest green). Shifts of the conductance versus voltage relationship to more negative potentials are plotted in a black to red gradient, with the brightest red indicating a shift of −16 mV. The pools that repeatedly destroyed membrane seals and were removed from further analysis are indicated by grey boxes. (C) Results of the fluorescence based portion of the screen presented as RPC for each of the 96 FOL pools plotted in a black to green color gradient with pools that had no effect on the tryptophan colored in black, and largest effects colored in bright green. Grey boxes indicate the pools that had a larger effect on free tryptophan fluorescence than the fluorescence of the protein.

In the first round of the electrophysiology screen, we applied 96 pools of seven to ten FOL compounds to inside-out patches at a concentration of 50 µM. The results from the electrophysiology-based screen are summarized in heat maps in figures 2A–B. Repeated application of four of the pools destroyed the membrane seals, and these pools were removed from further analysis (Figure 2A–B, grey boxes). The majority of the remaining FOL pools reduced the maximal conductance of EAG1-expressing patches (Figure 2A), and most of this conductance was recovered with washout. Specifically, out of 92 pools-analyzed, only two had no effect on the maximal conductance, 60 pools reduced the maximal conductance by 16–40% and 30 reduced the maximal conductance by >40%. 32 of the pools shifted the V_half_ of the conductance versus voltage plot by −1 to −15 mV, 19 pools did not evoke a shift, and 41 shifted the relationship to more positive potentials.

We sought to identify regulators that increased the open probability of the channel. As an initial screen for such channel regulators, the following criteria were used to identify ‘hit’ pools: 1) they facilitated channel opening by shifting the conductance versus voltage relationship to more negative potentials without evoking substantial channel block, 2) they slowed the deactivation time constant, and 3) the effects reversed with washout. To speed analysis, channel kinetics were analyzed only for pools that shifted the conductance versus voltage relationship to more negative potentials. Open channel blockers can elicit hyperpolarizing shifts in the conductance-voltage relationship and slowing of the deactivation time constant in proportion to the amount of block. Pools that evoked open channel block were excluded as hits. Using these criteria, five pools were identified as ‘hits’ in the electrophysiology portion of the screen. These five pools were separated into their individual components, and four compounds emerged as channel regulators. In addition, the fluorescence-based binding assay was performed on each of the hits from the electrophysiology portion of the screen.

In the first round of the fluorescence-based screen, pools of the FOL compounds, with each compound at 100 µM concentration, were applied to 4 µM Meag#505-702. Out of the 96 pools examined, twenty nine showed dramatically high fluorescence intensity and/or absorbance, precluding accurate measurement of the Meag#505-702 fluorescence in the presence of these compounds. The effect of these compounds on the fluorescence of mEAG1#505-702 was analyzed at 2- to 100-fold lower concentrations. Pools that had a larger effect on the fluorescence of free tryptophan in solution compared to the effect on the fluorescence of mEAG1#505-702 were removed from further analysis (Fig. 2C, grey boxes). The remaining pools were categorized based on their RPC. The results of the fluorescence based screen are summarized in Fig. 2C where the RPC of the 96 pools is plotted in a green to black color gradient with pools that had no effect on the fluorescence of the protein colored in black. Fifteen pools with a RPC of 10% or higher were identified as ‘hits’ and the individual compounds of these pools were examined. RPC of a pool represents a summation of RPC of individual compounds in the pool, therefore individual compound in the pool might have RPC lower than the total RPC of the pool. Out of the fifteen pools, seven contained individual compounds with RPC<10% and the remaining eight pools contained ten individual FOL compounds with RPC≥10%. The functional effects of these ten individual FOL compounds were also tested using electrophysiology.

**Figure 3:**
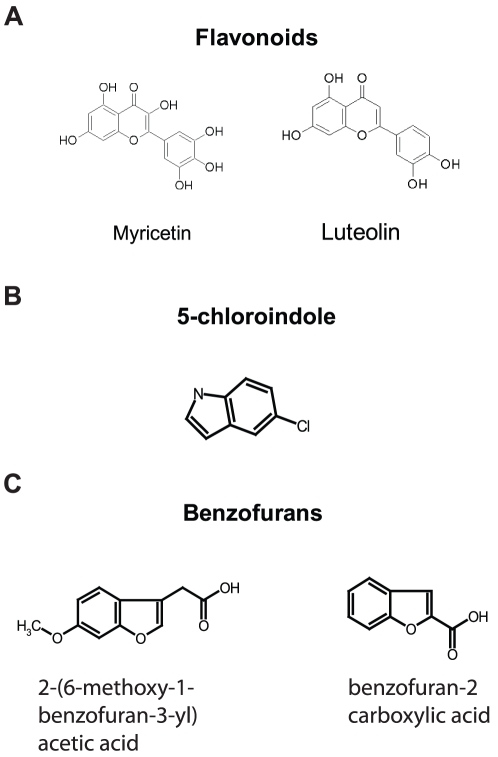
Chemical structures of the compounds identified as hits. (A) Structures of flavonoids luteolin and myricetin identified as EAG1 channel regulators by both electrophysiology and tryptophan fluorescence portions of the screen. (B) Structure of 5-chloroindole identified as EAG1 channel regulator by electrophysiology screen. (C) Structures of benzofurans 2-(6-methoxy-1-benzofuran-3-yl) acetic acid and benzofuran-2 carboxylic acid identified as binding partners of EAG1 channels by the tryptophan fluorescence portion of the screen.

As a result of this two-pronged screen several compounds emerged as binding partners and/or regulators of EAG1 channels (the chemical structures of the compounds are shown in Fig. 3). Two FOL compounds, luteolin and myricetin, were identified as regulators of EAG1 channels by both the electrophysiology and fluorescence arms of the screen. 5-chloroindole was identified as a ‘hit’ with the electrophysiology screen, but showed no relative percent change in the tryptophan fluorescence. This suggests that 5-chloroindole may not bind to the CNBD to alter channel gating. Conversely, the benzofurans 2-(6-methoxy-1-benzofuran-3-yl) acetic acid and benzofuran-2 carboxylic acid decreased the fluorescence intensity of mEAG1#505-702, but had either no effect on EAG1 channel gating or simply blocked the currents indicating that the binding of these ligands does not regulate EAG1 channels. We focused our attention on the two common hits identified by the combined screen and three non-common hits that showed the largest effects.

### Luteolin and myricetin bound to the CNBD region and modulated gating of EAG1 channels

**Figure 4:**
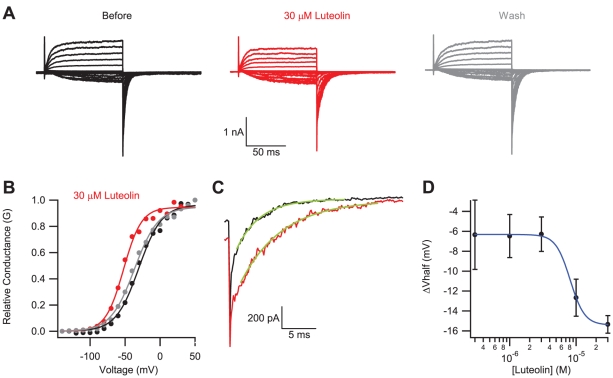
Luteolin modulated currents from EAG1 channels. Current traces (A) and conductance-voltage relationship (B) for EAG1 channels recorded in the inside-out patch configuration in the absence (black), presence (red) and after washout (grey) of 30 µM luteolin. (C) The tail current recorded at −120 mV, following a voltage step to −50 mV, in the absence (black) and presence (red) of 30 µM luteolin, fit with single exponentials to give time constants of 3.5 ms before, and 6.2 ms after application of luteolin. (D) Plot of the change in the V_half_ versus luteolin concentration, fit with a Hill equation with a binding affinity of 9.1 µM, and a Hill coefficient of 4.

Luteolin and myricetin, both flavonoids, were identified as binding partners and modulators of EAG1 channels. Both compounds had similar effects on the channel: they facilitated channel opening at more negative potentials and slowed deactivation in a concentration-dependent manner (Fig. 4A and Fig. S2A). 30 µM luteolin shifted conductance/voltage relationship by an average of −15±0.9 mV, N = 4, and increased deactivation time constant at −120 mV by 2.5 –fold from an average of 3.3±0.4 ms before, to an average of 8.3±1.9 with luteolin, N = 4 (Figs. 4B–C). Plots of the change in V_half_ versus luteolin concentration (Fig. 4D), and time constant of tail current deactivation versus luteolin concentration (not shown) were fit with the Hill equation. Luteolin binds to EAG1 with an EC_50_ of 8 µM and a Hill coefficient of 4, reflecting the binding of multiple flavonoids. At concentrations up to 30 µM, the effect of luteolin was almost completely reversible (Fig. 4A). Similar to luteolin, myricetin shifted the conductance/voltage relationship to facilitate channel opening at more hyperpolarized potentials (Fig. S2B), and slowed deactivation (Fig. S2C). At higher concentrations, both flavonoids blocked the current.

**Figure 5:**
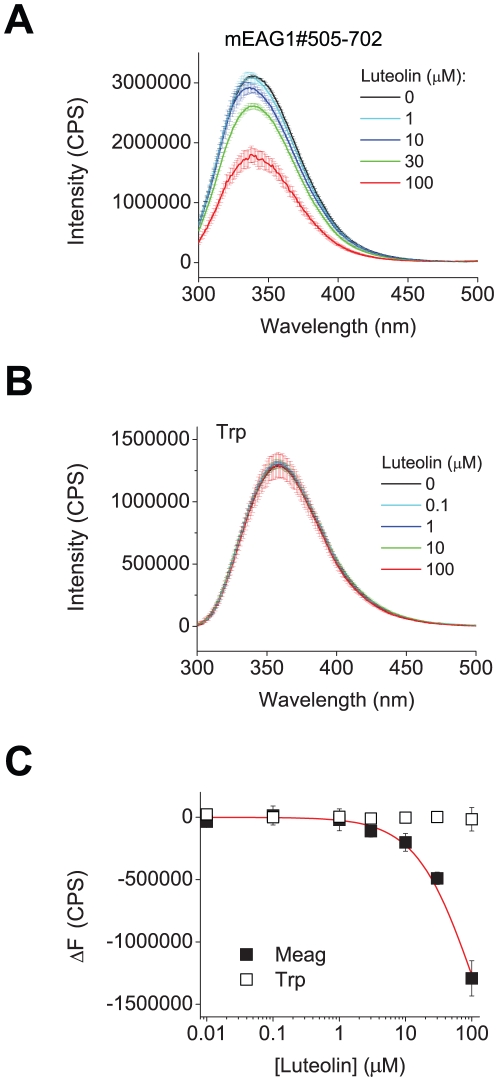
Luteolin reduced mEAG1#505-702 fluorescence in a concentration dependent manner. (A) The inner filter corrected, and background subtracted emission spectra of mEAG1#505-702 recorded without and with the indicated concentrations of luteolin. (B) The inner filter corrected, and background subtracted emission spectra of free tryptophan in solution recorded without and with the indicated concentrations of luteolin. (C) Plots of change in the peak emission fluorescence intensity versus total luteolin concentration for mEAG#505-702 (filled squares) and free tryptophan (open squares), fit with equation (5). The peak fluorescence intensity corresponded to fluorescence intensity at 338 nm for mEAG1#505-702 and at 357 nm for free tryptophan. The change in the peak fluorescence intensity was calculated by subtracting averaged peak emission intensity for low concentrations of luteolin (intensities at 0, 0.01 and 0.1 µM luteolin at 338 nm) from the peak emission intensities. The binding affinity of luteolin was ≥100 µM for mEAG1#505-702.

Luteolin and myricetin also decreased the fluorescence intensity of mEAG1#505-702 in a concentration dependent manner (Fig. 5A and Fig. S3A). Both compounds had little effect on the fluorescence of free tryptophan (Fig. 5B and Fig. S3B), indicating that the fluorescence change is specific to the flavonoid binding to mEAG1#505-702. To determine the biding affinity, we plotted the change in the peak fluorescence intensity versus the total concentration of luteolin (Fig. 5C) and myricetin (Fig. S3C). A fit to equation 5 (METHODS) indicated a binding affinity of ≥100 µM for both luteolin and myricetin. Both luteolin and myricetin have limited solubility in aqueous solutions and precipitate at concentrations higher than 100 µM. In addition, application of luteolin at concentrations higher than 100 µM and myricetin at concentrations higher than 30 µM irreversibly abolished currents, precluding a more in-depth characterization of these channel regulators with fluorescence or electrophysiology at higher concentrations.

The apparent binding affinity of the two flavonoids determined with electrophysiology is higher than the lower limit for the apparent binding affinity determined based on the changes in the fluorescence. This might indicate that the effects of flavonoids on channel gating and fluorescence originate from different binding sites/mechanisms. Alternatively, it could reflect a difference between binding to intact EAG1 channels and isolated C-terminal fragments. Conformational changes of the intact channel following ligand binding shape the apparent binding affinity determined with electrophysiology [20], therefore, the binding affinity of an isolated region might differ from that of the intact channel. Further, the apparent binding affinity determined with electrophysiology reflects cooperative ligand-binding to the full length tetrameric channel whereas the binding affinity determined with tryptophan fluorescence reflects ligand binding to a monomeric, isolated C-terminal domain and therefore lacks effects of cooperative interactions. The precise binding sites of the flavonoids remain to be determined.

### 5-Chloroindole modulated EAG1 channel gating but had no specific effect on the fluorescence of mEAG1#505-702

**Figure 6:**
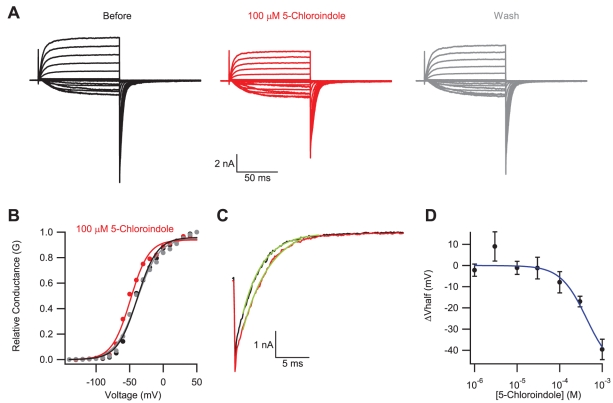
5-Chloroindole modulated currents from EAG1 channels. Current traces (A) and conductance-voltage relationship (B) for EAG1 channels recorded in the inside-out patch configuration in the absence (black), presence (red) and after washout (grey) of 100 µM 5-chloroindole. (C) The tail current at −120 mV, following a voltage step to −50 mV, in the absence (black) and presence (red) of 100 µM 5-chloroindole, and fit with single exponentials to give time constants of 3.0 ms before, and 4.1 ms after application of 5-chloroindole. (D) Plot of the change in the V_half_ versus 5-chloroindole concentration, fit with a Hill equation. The binding affinity and Hill coefficient of 5-chloroindole were 420 µM and 1.4 respectively.

Similar to the flavonoids, 5-chloroindole facilitated opening of EAG1 channels at more hyperpolarizing potentials (Fig. 6). 100 µM 5-chloroindole shifted the V_half_ of the conductance versus voltage relationship by an average of −8±5 mV, N = 3 (Fig. 6B). Application of 100 µM 5-chloroindole also slowed the deactivation time constant from 3.6±0.5 ms to 6.3±1.2 ms, N = 3 (Fig. 6C). Application of 5-chloroindole at concentrations higher than 1 mM abolished the currents, yet the concentration-response curve has not saturated at this concentration (Fig. 6D). Therefore, the apparent binding affinity of 420 µM estimated by fitting the concentration versus response plot with a Hill equation represents a lower limit.

Application of 5-chloroindole decreased the fluorescence of mEAG#505-702 to the same extent as the fluorescence of free tryptophan in solution (not shown), indicating that the effect on the tryptophan fluorescence is due to non-specific quenching. The absence of a specific change in tryptophan fluorescence suggests that 5-choloroindole modulates EAG1 channels through binding to regions other than the C-terminal domain and/or the binding of the 5-chloroindole to the C-terminal domain is not detected by the tryptophan fluorescence.

### Benzofurans bound to the C-terminal region but did not facilitate opening of EAG1 channels

**Figure 7:**
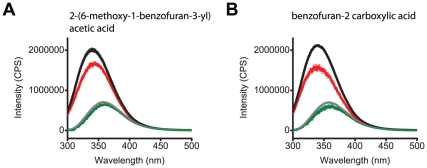
2-(6-methoxy-1-benzofuran-3-yl) acetic acid and benzofuran-2 carboxylic acid reduced fluorescence of mEAG1#505-702. The inner filter corrected, and background subtracted emission spectra of mEAG1#505-702 recorded without (black) and with (red) 30 µM 2-(6-methoxy-1-benzofuran-3-yl) acetic acid (A), and 50 µM benzofuran-2 carboxylic acid (B). Also shown are the inner filter corrected, and background subtracted emission spectra of free tryptophan recorded without (grey) and with (green) 30 µM 2-(6-methoxy-1-benzofuran-3-yl) acetic acid (A) and 50 µM benzofuran-2 carboxylic acid (B).

The benzofurans 2-(6-methoxy-1-benzofuran-3-yl) acetic acid and benzofuran-2 carboxylic acid decreased the fluorescence intensity of mEAG1#505-702 but had little effect on fluorescence intensity of free tryptophan (Fig. 7). At 100 µM, both compounds were fluorescent upon excitation with 280 nm and showed fluorescence intensity several times higher than the intensity of mEAG1#505-702. Therefore, to determine the effect of the compounds on the fluorescence of mEAG1#505-702, the concentration was decreased to 30 µM for 2-(6-methoxy-1-benzofuran-3-yl) acetic acid and 50 µM for benzofuran-2 carboxylic acid (Fig. 7).

The benzofurans did not affect channel function, they did not shift the conductance versus voltage relationship or slow the deactivation time course. The observation that the benzofurans do not facilitate channel opening suggests that binding of these compounds to the C-terminal domain does not evoke conformational changes that regulate EAG1 channel opening.

## Discussion

Here we employed the FOL library, a collection of small natural metabolites and fragments thereof, to identify ligands for EAG1 channels with a novel, two-pronged electrophysiology and fluorescence-based screen. This combination of two orthogonal methods provides a label-free approach to screen ion channels with chemical libraries. Recording currents from inside-out patches is the most direct approach to determine functional effect of compounds on ion channels with intracellular ligand binding motifs. Furthermore the fluorescence arm of the screen can be used to identify ligands of soluble proteins or fragments that cannot be characterized functionally with electrophysiology. The tryptophan fluorescence screen does not require development of a fluorescent or radio-labeled ligand, has a high signal- to-noise ratio, and could be easily automated for a high-throughput format. Used together, the two arms of the screen overcome limitations of each individual portion. For example, the presence of pore blocking compounds in the FOL library pools could obscure detection of channel regulators in the electrophysiology arm of the screen. The fluorescence arm of the screen, however, would detect binding partners in pools containing pore blockers. Conversely, the binding of some FOL compounds might not evoke changes in tryptophan fluorescence, but their functional effects might be picked up by the electrophysiology arm of the screen.

Screening chemical libraries is an efficient way to uncover novel ligands and has moved to the forefront of drug discovery following the development of large chemical libraries and high-throughput screening techniques [21], [22], [23], [24], [25]. There are several problems with the currently available screening techniques that we avoid here. For example, most high-throughput screens assay competitive binding between a known ligand, labeled with a fluorescent or radioactive tag, and the small molecules to be screened. These screens therefore require prior knowledge of a ligand, precluding their use for our study. Labeling known ligands can also present complications. The hazardous nature of radioactivity makes the radioactive screens costly and less popular [21], [26]. The non-radioactive methods require either fluorescent-labeling or immobilization of ligand and/or receptor. Fluorescence labeling and immobilization of ligand and/or receptor can disturb the ligand binding affinity and receptor integrity. Development of fluorescent ligands can also be difficult. In order to be useful, the fluorescent ligands should have high quantum yield, high extinction coefficient, and small size [21].

Screening libraries against membrane proteins, including ion channels, presents further challenges. The multi-subunit nature of ion channels and presence of membrane spanning domains makes surface immobilization based assays difficult [21]. Screens designed specifically for ion channels typically use voltage- or calcium-sensitive fluorescent dyes or non-radioactive tracer ions [27]. These indirect methods detect global changes that might not be specific to the channel of interest. To overcome these difficulties we combined a direct functional assay based on electrophysiology with a direct binding assay based on label-free tryptophan fluorescence.

Using the two-pronged screen, we identified several novel ligands of EAG1 channels. Luteolin and myricetin, both flavonoids, emerged from the screen as regulators of EAG1 channels that facilitated channel opening and slowed deactivation. They also decreased tryptophan fluorescence of the isolated C-terminal region, indicating binding to this domain. Flavonoids are plant metabolites that are acquired by mammals through dietary intake. Both luteolin and myricetin are present in fruits and vegetables consumed by humans, including grapes, berries, green peppers, and olive oil. Compounds in the flavonoid family have been previously shown to inhibit currents from another EAG family channel, hERG1 [28], [29], [30], [31]. For the flavonoid hesperetin, this inhibition has an EC_50_ of 267 µM, and was mediated by binding to a site in the hERG1 channel pore [30]. It should be noted, however, that myricetin did not inhibit hERG1 channels, and luteolin was not examined [31]. The isoflavonoid genistein facilitated opening of HCN1 and HCN2 channels, and this facilitation of channel opening persisted in HCN1 channels lacking only their CNBD, but not in channels missing the entire carboxy-terminal domain including the C-linker [32]. While the goal of our study was to develop a novel screen to identify ligands of EAG1 channels, future investigation is necessary to determine whether the binding detected by the fluorescence portion of the screen is responsible for the functional effects of the flavonoids revealed by electrophysiology.

In addition to the flavonoids, 5-chloroindole was shown to facilitate EAG1 channel opening. Whereas 5-chloroindole is not a natural compound, it is a derivative of tryptophan and a structural mimic of serotonin. The compound did not evoke changes in the fluorescence of the isolated CNBD domain, suggesting that either the binding of the compound was not detected by the fluorescence assay and/or the indole regulates EAG1 channels through regions other than the CNBD. One of the potential regions of action could be the amino-terminal domain of EAG family channels that contains a PAS domain known to serve as a ligand binding domain in other proteins [14]. Application of both flavonoids and 5-chloroindole at high concentrations abolished the currents, suggesting that these compounds may be acting on site(s) in the channel other than the intracellular domains or/and are affecting channel behavior through a non-specific site(s) in the cell membrane. Finally, benzofurans, metabolites synthesized in plants but absent in human diet, were shown to bind to the carboxy- terminal region of EAG1 channel. They did not facilitate currents from EAG1 channels similar to the reports of the absence of hERG1 channel facilitation by other compounds from the benzofuran family [33], [34], [35].

Flavonoids, benzofurans, and indoles are abundant in nature and have a wide range of physiological functions, including antioxidative protection, anti-tumor and anti-inflammatory activity, and regulation of blood pressure and neurotransmitter release [36], [37], [38]. The discovery of ligands from diverse chemical families might be useful for development of new EAG1 specific pharmaceutical agents. These ligands could be used as prototypes of EAG1 specific drugs. Knowledge of the ligands also opens up new possibilities for in-depth study of the regulatory mechanisms of EAG1 channels.

## Materials and Methods

### Preparation of the pools of FOL compounds

The FOL library was assembled from small biological metabolites or derivatives thereof, and molecules with surface features and solubility properties of cellular metabolites. The selected 768 small molecules (the latest version of the FOL library contains over 1400 compounds) were chosen from evolutionary diverse organisms and had molecular weight of less than 350 as previously described [18]. The FOL compounds were distributed into a 96 well plate with each well containing 7–10 individual compounds of dissimilar shape and dissolved in 20 µl of methanol at a concentration of 6 mM. To prevent possible damage to patches, the methanol was evaporated with a speed vacuum for 20 minutes following centrifugation of the 96 well plates at 1000×rpm for 20 minutes. After visual inspection indicated that all methanol had evaporated, pools were re-suspended in 20 µl of DMSO, transferred to conical screw-cap tubes and stored at −20°C.

### Electrophysiology

The cDNA encoding mEAG1 channels in pGH19 vector was kindly provided by G. Robertson (University of Wisconsin-Madison, Madison, WI). The cRNA was transcribed using the T7 mMessage mMachine kit (Ambion). *Xenopus laevis* oocytes were defolliculated and injected with the cRNA as previously described [39]. Following manual removal of the vitelline membrane, currents were recorded in the inside-out patch configuration [40] with an EPC-10 patch-clamp amplifier (HEKA Electronik). Data were acquired with Pulse software (HEKA Elektronik) and analyzed with Igor (WaveMetrics, Inc). Patch pipettes were pulled from borosilicate glass and had resistances of 0.40–1 MΩ after fire polishing. The intracellular (bath) and extracellular (pipette) solutions contained 130 mM KCl, 10 mM HEPES, 0.2 mM EDTA, pH 7.2. The pools of individual FOL compounds were added to the bath solution as indicated. After channel regulators were identified, they were applied to patches with the RSC-100 solution changer (BioLogic). The mEAG1 currents were elicited by applying a series of 0.1 s voltage pulses (ranging from −140 to +50 mV in 10 mV increments) from a holding potential of −100 mV, followed by a 0.5 s voltage pulse to −120 mV. Currents were not leak subtracted.

To obtain conductance versus voltage curves for all electrophysiology experiments, peak tail current amplitudes at −120 mV, were normalized to the largest peak conductance amplitude, which followed a step to +50 mV. These normalized data were then plotted against the test voltage, and were fit with a Boltzmann equation:
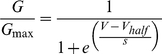
(1)Where V represents the test voltage (mV), V_half_ is the midpoint activation voltage (mV), and s is the slope of the relation (mV).

The dose response relationships for both ÄV_half_ and the deactivation time constants were fit with a Hill equation:
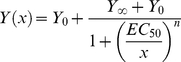
(2)Where Y_0_ represents the minimum response and Y_∞_ the maximum, EC_50_ represents the binding affinity, and *n* the Hill coefficient.

### Application of FOL library to inside-out patches

In the first round of screening the pools of FOL compounds were applied at 50 µM concentration to inside-out patches expressing mEAG1 channels. Each of the 96 pools was applied to three separate patches and each patch was treated with only one pool. Changes in the current amplitude, kinetics, and voltage dependence were used to discern the modulators of channel activity. Once the ‘hit’ pools were identified, they were further studied by applying the individual components to inside-out patches at a 100 µM concentration. Similar to the application of the FOL pools, the individual compounds were applied to three separate patches with each patch treated with only one compound.

Due to the limited supply of the FOL library pools, we used RC-28 chamber (Warner Instruments) optimized for small volumes. To avoid contamination between different pools, the chamber was thoroughly cleaned after each application with a 40 mM Na_3_PO_4_ solution, then rinsed with 1% HCl, and the bottom cover-slip was replaced. To further avoid cross pool contamination, all tubing and syringes were exposed to only one pool or individual compound.

### Protein expression and purification

The C-terminal region of the mEAG1 channel containing the CNBD (mEAG1#505-702) was expressed in BL21 (DE3) cells as described in Brelidze et al. (2009). Briefly, the DNA encoding mEAG1#505-702 was subcloned into the pETGQ vector [41] and transformed into BL21 (DE3) cells. The cells were grown at 37°C to the OD of 0.6–0.8 and induced subsequently with 1 M IPTG. After growing overnight at 18°C, the cells were harvested by centrifugation at 5000×rpm for 15 min at 4°C. The cell pellet was frozen at −80°C until further use. To purify mEAG1#505-702, the frozen cell pellet was resuspended in a lysis buffer (150 mM KCl, 10% Glycerol, 1 mM TCEP, 30 mM HEPES, 1 mM PMSF and 2.5 mg/ml DNase; pH 7.5) and lysed in an Emulsiflex - C5 (Avestin). Insoluble protein was separated by centrifugation for 45 min at 40,000×rpm at 4°C in a Beckman 45Ti rotor. The mEAG1#505-702 was then purified from the supernatant by Ni^2+^ - NTA chromatography and eluted on a linear gradient to 500 mM imidazole. The 6×His tag was cleaved with thrombin protease (Calbiochem) and separated with size exclusion chromatography on a Superdex 200 column (Amersham Biosciences) equilibrated with the buffer used for the subsequent experiments (150 mM KCl, 10% Glycerol, 1 mM TCEP, 30 mM HEPES; pH 7.5). The purified protein was stored at −80°C in small aliquots and thawed immediately before the experiments. Protein concentration was determined by absorbance at 280 nm, and used at a concentration of 4 µM.

### Fluorescence measurements

Fluorescence intensity was recorded with Fluorolog 3 spectrophotometer (HORIBA, Jobin Yvon) using FluorEssence software. The sample was placed in a 100 µM chamber of a quartz cuvette, excited at 280 nm, and the emission spectrum was recorded from 300 to 500 nm. In the first round of screening the pools of the FOL compounds were applied at 100 µM concentration to 4 µM mEAG1#505-702 and 4 µM free tryptophan in solution. To account for the decrease in excitation and emission intensities due to the optical density, observed fluorescence intensities of the sample were corrected for the inner filter effect according to the equation [19]:
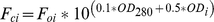
(3)


Where F_ci_ and F_oi_ represent the corrected and observed fluorescence intensities at i nm wavelength, and OD_280_ and OD_i_ are the absorbance recorded at 280 nm and i nm wavelength respectively. To calculate the final fluorescence intensity in the presence of an FOL compound, the corrected intensity of the FOL compound alone was subtracted from the corrected sample intensity. Each experiment was repeated at least three times. Optical density of 4 µM mEAG1#505-702 at 280 nm is relatively low. Thus, to simplify the analysis in the first round of the screen, we only corrected the inner filter effect due to the optical density of the FOL compounds. Once the ligands were identified, the fluorescence intensities used to estimate their binding affinity were corrected for the inner filter effect due to the optical density of both the FOL compound and mEAG1#505-702.

To estimate the binding affinity, plots of the change of the peak fluorescence intensities versus total FOL compound concentration were analyzed as in Brelidze et al. (2009) and Cukkemane et al. (2007).
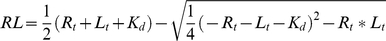
(4)

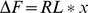
(5)


Where R, L, and RL are concentrations of the free receptor and ligand, and receptor-ligand complex, respectively; R_t_ and L_t_ are total receptor and ligand concentrations; K_d_ is the ligand binding affinity; ΔF is the peak fluorescence change, and x is a scaling factor.

The data analysis and fitting of the plots was performed in Origin (Microcal Software, Inc). The error bars on the figures correspond to the SEM.

## Supporting Information

Figure S1 Example of data analysis for the tryptophan fluorescence portion of the screen. (A) In each experiment, emission spectra of mEAG1#505-702 were recorded in the absence (black line), and presence (red line) of a FOL pool. In parallel, the fluorescence spectra of free tryptophan were recorded in the absence (grey trace) and presence (green trace) of the FOL pool. Emission spectra of the FOL pool alone was recorded (blue line) and subsequently subtracted from the spectra of mEAG1#505-702 and free tryptophan recorded in the presence of the pool, to correct for the background fluorescence. (B) The absorbance spectrum of the FOL pool was recorded to correct for the inner filter effect. (C) The inner filter corrected, and background subtracted emission spectra of mEAG1#505-702 in the absence (black line) and presence (red line) of the FOL pool, and similarly analyzed free tryptophan emission spectra in the absence (grey line) and presence (green line) of the FOL pool.
(0.37 MB PDF)

Figure S2 Myricetin modulated currents from EAG1 channels. Current traces (A) and conductance/voltage relationship (B) for EAG1 channels recorded in the inside-out patch configuration in the absence (black) and presence (red) of 10 or 100 µM myricetin, as indicated. (C) The tail current recorded at −120 mV, following a voltage step to −50 mV, in the absence (black) and presence (red) of 100 µM myricetin, fit with single exponentials to give time constants of 3.7 ms before, and 7.2 ms after application of myricetin.
(0.83 MB PDF)

Figure S3 Myricetin reduced mEAG1#505-702 fluorescence in a concentration dependent manner. (A) The inner filter corrected, and background subtracted emission spectra of mEAG1#505-702 recorded without and with the indicated concentrations of myricetin. (B) The inner filter corrected, and background subtracted emission spectra of free tryptophan in solution recorded without and with the indicated concentrations of myricetin. (C) Plots of change in the peak emission fluorescence intensity versus total myricetin concentration for mEAG#505-702 (filled squares) and free tryptophan (open squares), fit with equation (5). The peak fluorescence intensity corresponded to fluorescence intensity at 338 nm for mEAG1#505-702 and at 357 nm for free tryptophan. The change in the peak fluorescence intensity was calculated by subtracting averaged peak emission intensity for low concentrations of luteolin (intensities at 0, 1 and 3 µM myricetin at 338 nm) from the peak emission intensities. The binding affinity of myricetin is >100 µM for mEAG1#505-702.
(0.40 MB PDF)
