# Supplementary figures and images for: Identifying Regulators for EAG1 Channels with a Novel Electrophysiology and Tryptophan Fluorescence Based Screen
Source: PLoS One. 2010 Sep 2;5(9):e12523. doi: 10.1371/journal.pone.0012523 (PMC2932742; doi:10.1371/journal.pone.0012523)

**A**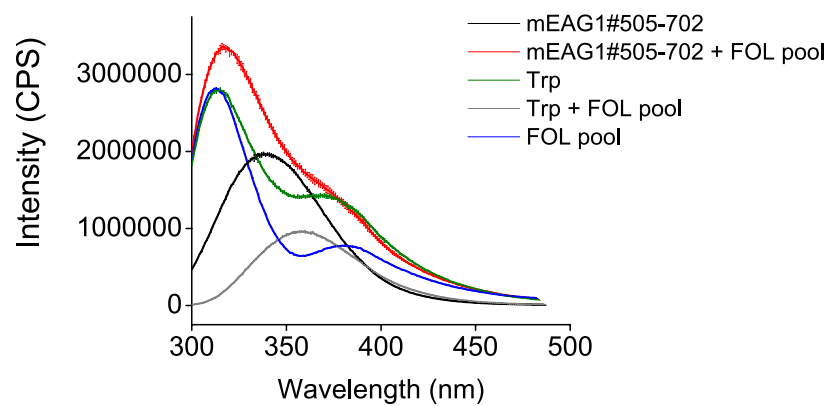**B**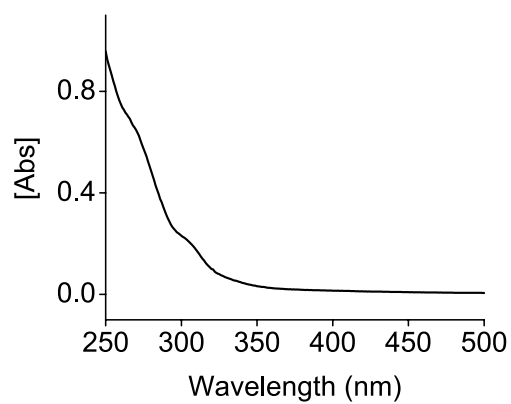**C**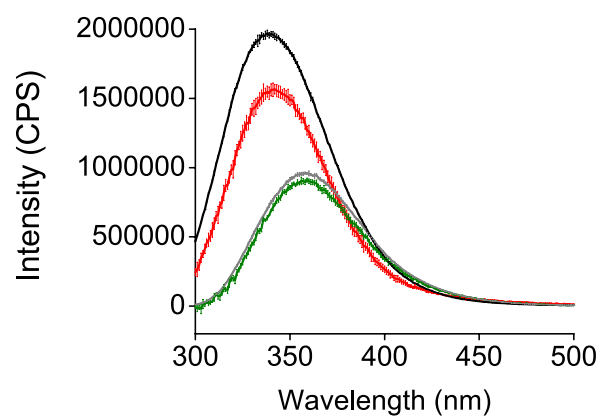

Supplement: Figure S1 — Example of data analysis for the tryptophan fluorescence portion of the screen. (A) In each experiment, emission spectra of mEAG1#505-702 were recorded in the absence (black line), and presence (red line) of a FOL pool. In parallel, the fluorescence spectra of free tryptophan were recorded in the absence (grey trace) and presence (green trace) of the FOL pool. Emission spectra of the FOL pool alone was recorded (blue line) and subsequently subtracted from the spectra of mEAG1#505-702 and free tryptophan recorded in the presence of the pool, to correct for the background fluorescence. (B) The absorbance spectrum of the FOL pool was recorded to correct for the inner filter effect. (C) The inner filter corrected, and background subtracted emission spectra of mEAG1#505-702 in the absence (black line) and presence (red line) of the FOL pool, and similarly analyzed free tryptophan emission spectra in the absence (grey line) and presence (green line) of the FOL pool. (0.37 MB PDF) [file pone.0012523.s001.pdf]

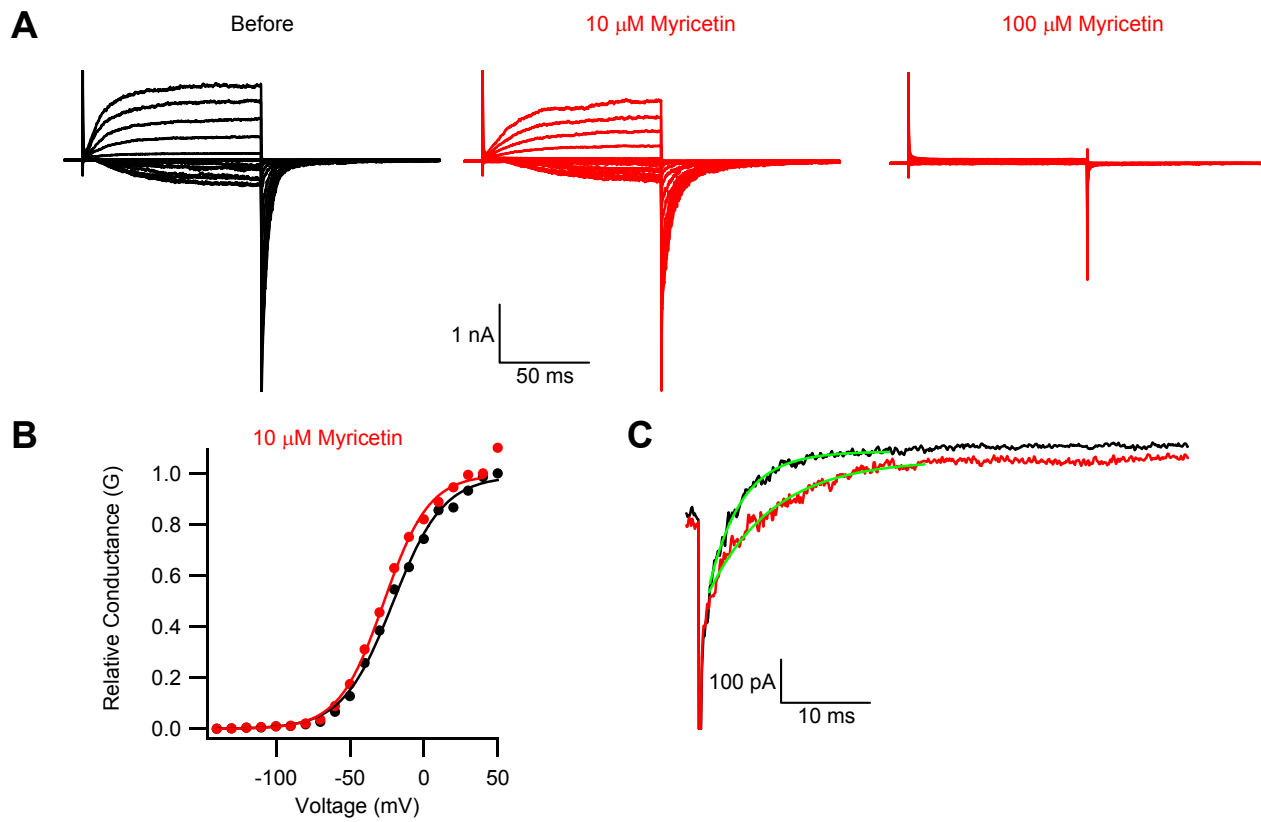

**Supplementary Fig. 2**

Supplement: Figure S2 — Myricetin modulated currents from EAG1 channels. Current traces (A) and conductance/voltage relationship (B) for EAG1 channels recorded in the inside-out patch configuration in the absence (black) and presence (red) of 10 or 100 µM myricetin, as indicated. (C) The tail current recorded at −120 mV, following a voltage step to −50 mV, in the absence (black) and presence (red) of 100 µM myricetin, fit with single exponentials to give time constants of 3.7 ms before, and 7.2 ms after application of myricetin. (0.83 MB PDF) [file pone.0012523.s002.pdf]

**A**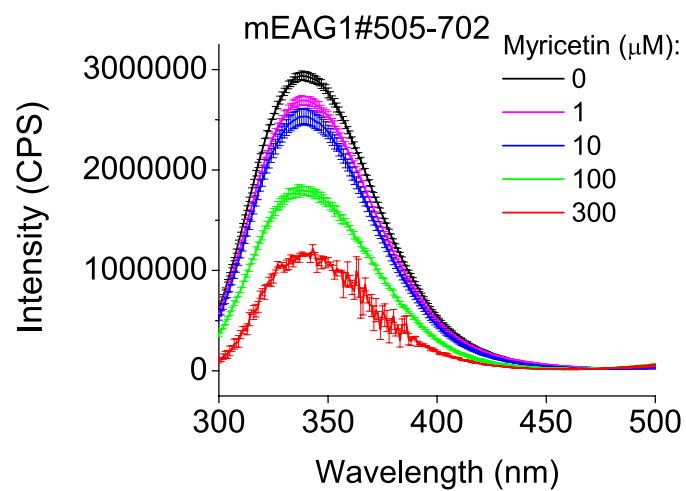**B**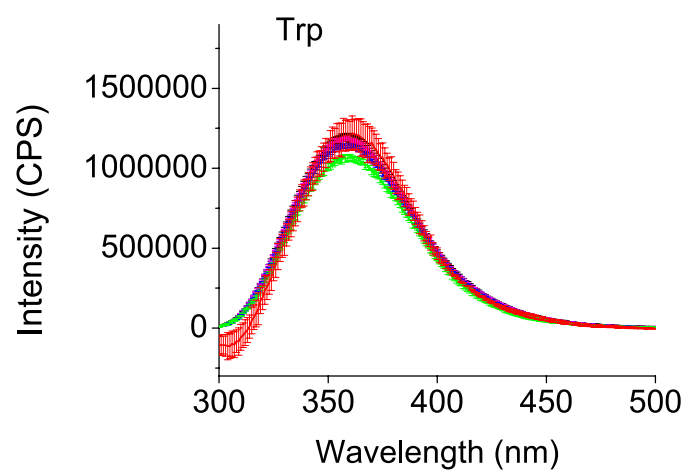**C**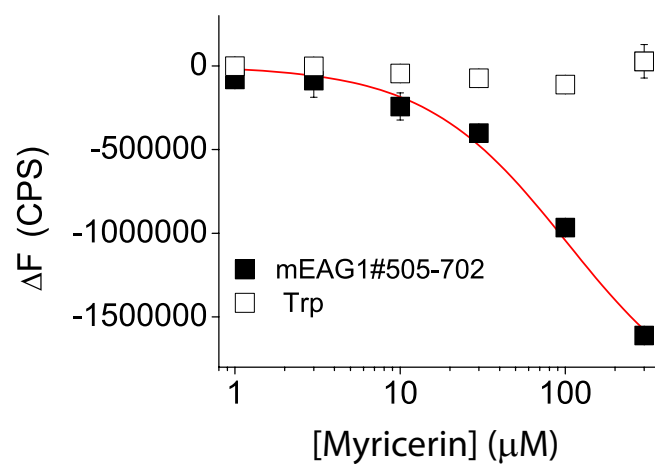**Supplementary Fig. 3**

Supplement: Figure S3 — Myricetin reduced mEAG1#505-702 fluorescence in a concentration dependent manner. (A) The inner filter corrected, and background subtracted emission spectra of mEAG1#505-702 recorded without and with the indicated concentrations of myricetin. (B) The inner filter corrected, and background subtracted emission spectra of free tryptophan in solution recorded without and with the indicated concentrations of myricetin. (C) Plots of change in the peak emission fluorescence intensity versus total myricetin concentration for mEAG#505-702 (filled squares) and free tryptophan (open squares), fit with equation (5). The peak fluorescence intensity corresponded to fluorescence intensity at 338 nm for mEAG1#505-702 and at 357 nm for free tryptophan. The change in the peak fluorescence intensity was calculated by subtracting averaged peak emission intensity for low concentrations of luteolin (intensities at 0, 1 and 3 µM myricetin at 338 nm) from the peak emission intensities. The binding affinity of myricetin is >100 µM for mEAG1#505-702. (0.40 MB PDF) [file pone.0012523.s003.pdf]
